# Relief Zones Enhance the Durability of Ultrathin Membranes in Electrochemical Conversion Devices

**DOI:** 10.1021/acsaem.5c03843

**Published:** 2026-02-04

**Authors:** Audrey K. Taylor, Megan McVeigh, Catherine Weiss, Kenneth C. Neyerlin

**Affiliations:** † Chemistry and Nanoscience Center, National Laboratory of the Rockies, Golden, Colorado 80401, United States; ‡ Energy Technologies Area, Lawrence Berkeley National Laboratory, Berkeley, California 94720, United States

**Keywords:** polymer electrolyte membrane, durability, accelerated stress test, mechanical failure, degradation, electrochemical conversion

## Abstract

Premature failures in electrochemical conversion systems often result when membrane electrode assemblies (MEAs) use ultrathin (≤15 μm-thick) polymer electrolyte membranes, susceptible to mechanical degradation from stress concentrations arising from device-level integration. Herein, relief zones were developed to mitigate mechanical degradation by alleviating excess and nonuniform compression across active areas. Relief zones, created through ablation of carbonaceous diffusion media, enable seamless adaptation across MEA dimensions without need for hardware modifications. Demonstrated using fuel cells as a case study, accelerated stress tests revealed a 6-fold lifetime improvement (∼1500 h) compared to conventional edge-protected MEAs, decoupling device-level engineering effects from material limitations.

## Graphical abstract

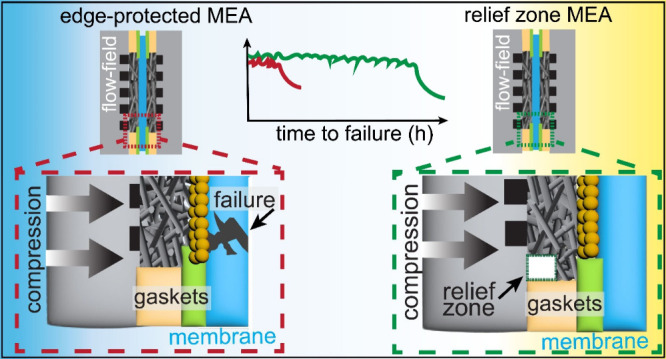

State-of-the-art polymer electrolyte membranes (PEMs) used in electrochemical systems have moved toward ultrathin dimensions (i.e., ≤15 μm-thick) to reduce cost and improve performance.^1,2^ At these ultrathin dimensions, however, trade-offs arise from increased reactant crossover and compromised mechanical stability.^3^ In particular, polymer electrolyte membrane fuel cells (PEMFCs) benefit from ultrathin PEMs in the membrane electrode assembly (MEA), positioning them for deployment in heavy-duty transport and stationary power applications among other emerging uses.^4^ Total cost of ownership analyses suggest that thicker membranes could be more cost-effective compared to thinner PEMs since refueling would be less frequent. This benefit becomes more significant for vehicles maintained over long durations (e.g., 15 yrs).^5^ Nevertheless, cost analyses indicate an optimal thickness between 10 and 12 μm, since lowered protonic resistances and higher power densities can be achieved, thereby decreasing the number of cells in a stack.^6^ Coordinated material development efforts aimed at stabilizing and mitigating degradation in ultrathin PEMs are relevant to a range of electrochemical conversion systems; thus, assembly or packaging strategies also have broad applicability regardless of technology type.

Chemical design strategies that improve transport properties are often linked to reduced mechanical stability. Entropic penalties associated with increased hydration of ionic domains pose challenges to the dimensional stability of PEMs with high ion exchange capacities. Lessening such trade-offs have included the use of reinforcement layers, which minimize in-plane dimensional changes.^7^ Although these approaches can reduce mechanical degradation, their efficacy depends on an appropriate integration of the PEM within not only the MEA but also the single-cell or stack hardware in which it is to be examined.

The architecture of the MEA and its integration within the cell hardware can impact the occurrence of premature failures in the PEM. Nonideal component integration,^8^ including both material defects from processing^9,10^ and stresses generated from compression during cell assembly,^10^ can lead to early mechanical degradation.^11^ These irregularities often go undetected during routine performance assessments without specifically sensitive testing conditions.^12^ Premature degradation events, identified as pin-holes, cracks, and/or tears, are most prevalent in PEMs with ultrathin dimensions,^13^ and although reinforcement layers mitigate dimensional changes from water uptake, they do not prevent or resolve these integration-related challenges.

Subgaskets (SGs) placed between the PEM and the gas diffusion layer (GDL) or porous transport layer (PTL) have traditionally been used to protect the PEM from irregularities and local stress concentrations along the sealing perimeter.^14,15^ Dimensionally stable SGs are used to limit interfacial deformation of the soft PEM with relatively stiff materials and to limit buckling within voids upon water uptake.^16,17^ These so-called “edge-protected MEAs” have demonstrated improved durability compared to those without, as well as for PEMs ≥25 μm in thickness.^18,19^ These conventional edge-protected MEAs rely on compression gaskets (CGs) for hard-stop sealing along the cell perimeter (Figure 1a). The resulting thickness of the CG within the assembled hardware specifies the compression target of the GDL (or PTL), ultimately affecting device performance and durability,^20–22^ whereas the SG extends to mask the CG-to-GDL interface with the PEM, providing additional mechanical support. Thin SGs (≤45 μm) ensure mechanical loading is focused onto CGs for adequate sealing. Increased pressure from excessive GDL compression along the SG regions was, however, found to be a primary contributor to early mechanical degradation in conventional edge-protected MEAs. This limitation underscores the need to revise current MEA designs to isolate intrinsic material properties from those related to integration and device engineering to achieve long-term operation and reproducible durability outcomes for ultrathin PEMs. To address these shortcomings, a novel MEA architecture is proposed to enable robust integration of ultrathin PEMs broadly applicable to electrochemical conversion systems.

**Figure 1:**
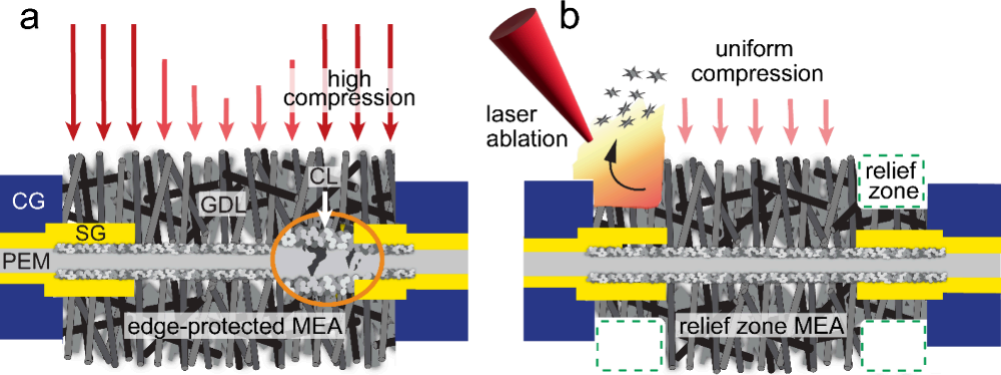
Schematics of the conventional edge-protected membrane electrode assembly (MEA) and the design concept for the relief zone MEA. The (a) edge-protected MEA utilizes a subgasket (SG, yellow) to stabilize the membrane from stresses along the MEA perimeter. The relief zone strategy in (b) is prepared using a raster laser ablation of the gas diffusion layer (GDL) along the overlap regions with the SG. Sealing of the MEA in both cases is achieved using compression gaskets (CGs, blue).

In this letter, we propose a relief zone strategy to alleviate stress from mechanical overcompression prevalent in MEAs. The relief zone mitigates mechanical degradation and promotes a uniform compression across the active area, enabling failure assessments strictly related to the chemical and mechanical properties of the PEM in question.^13^ Laser ablation was used to remove diffusion media along the exposed SG regions, leaving behind an intact microporous layer (MPL) as depicted in Figure 1b. The relief zone depth should be proportional to or preferably greater than the change in thickness specified by the compression target of the GDL (Equation S1). Cross-sectional and top-down images of the relief zone MEA are provided in Figure S1a-b. The relief zone approach is fully customizable, enabling ease of modification to a range of electrode component dimensions and material thicknesses without the need for costly hardware modifications. Thus, the use of relief zones is a promising approach for integration of ultrathin, state-of-the-art PEMs across electrochemical conversion systems that utilize zero-gap, MEA designs at specified compressions.

Spatial pressure distributions were assessed to examine stress points across the MEA within the single-cell hardware. Three-dimensional reconstructions of the surface contact pressure distributions are shown in Figure 2a-b for the edge-protected only and relief zone MEA, respectively. These data include the inlets of a quad serpentine land-channel array with CG and edge-protected SG regions. Figure 2a exhibits a variable pressure distribution across the CG edge with high mechanical loading concentrated along the SG and the flow-field lands. In contrast, the relief zone MEA shows a higher mechanical loading at the CG, ensuring a leak-free cell assembly and lessened pressure across the lands (Figure 2b). The pressure drop between the CG and the flow-field are indicative of the relief zone, which mitigates overcompression of the GDL along the lands. These results highlight the ability of relief zones to redistribute mechanical loading more effectively than edge-protection alone, establishing a direct link between contact pressure uniformity with implications to MEA durability and performance.

**Figure 2:**
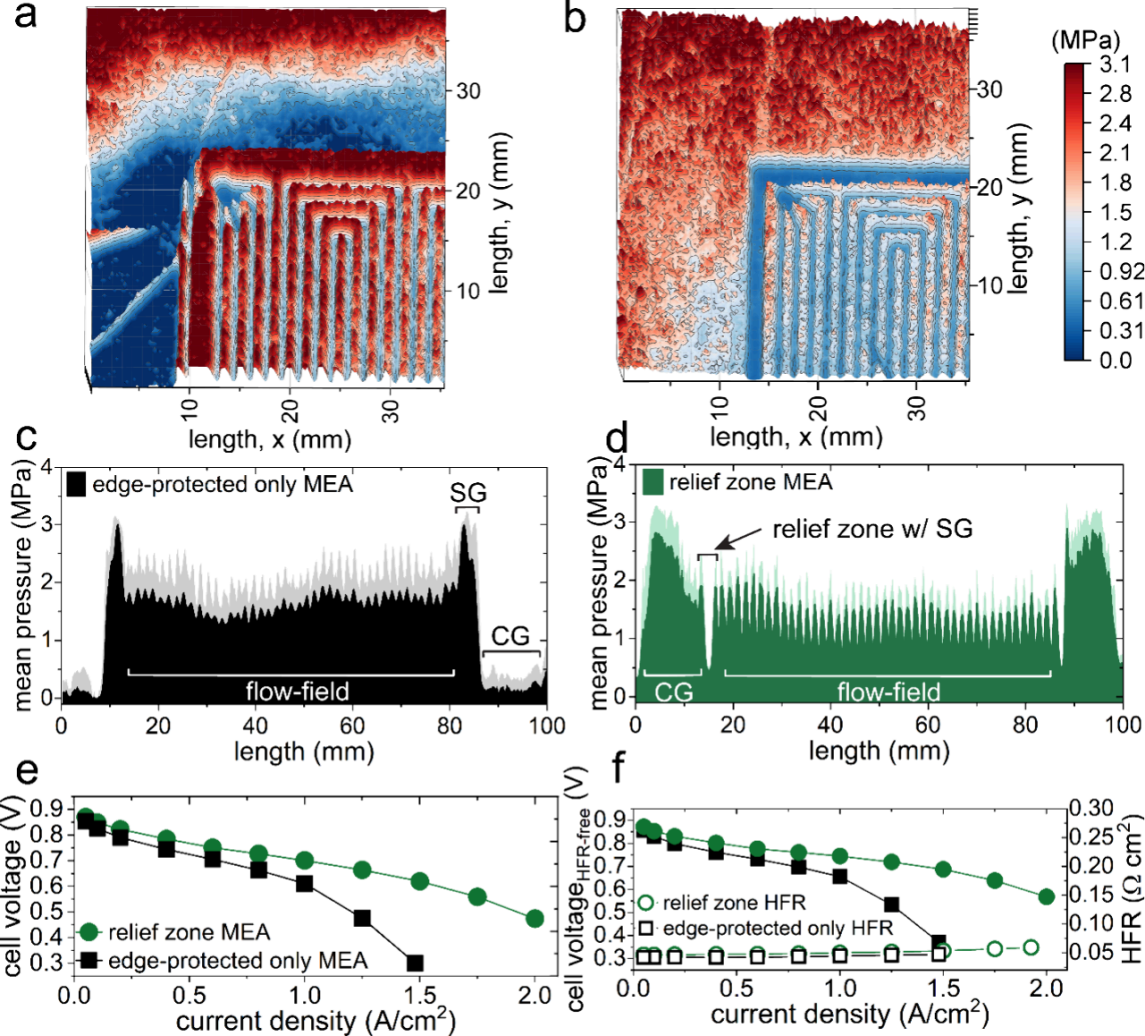
Three-dimensional (3D) surface reconstructions of the pressure distributions located at the flow-field inlets for the (a) edge-protected only and (b) relief zone MEAs and the associated mean pressure profiles showing the direction orthogonal to the land-channel paths for (c) and (d), respectively. The shaded regions in (c, d) represent one standard deviation from the mean pressure values. Polarization curves acquired in H_2_/air at 80 °C, 100% RH, and 150/150 kPa for the (e) relief zone and edge-protection only MEAs and the (f) corresponding high frequency resistance (HFR) corrected cell voltages.

Mean pressure profiles across the MEAs are shown in Figure 2c-d. The edge-protected only MEA exhibits a considerable concentration of pressure along the SG and a higher pressure and deviation across the flow-field (Figure 2c), whereas the relief zone MEA shows the highest pressures along the CG. The pressure drop at the SG indicates the presence of relief zones, which alleviates excess GDL compression, enabling a uniform pressure distribution (Figure 2d). These data agree with the selected reconstructions, and the pressure contact films are provided in Figure S2a-b. In short, regions of overcompression create irregular pressure concentrations and gradients that result in mechanical fatigue in addition to limited electrochemical performance.

Polarization data from the relief zone MEAs exhibit a ∼16 mV decrease in kinetic overpotential (Figure 2e-f). This small kinetic offset could indicate differences in the local concentration of O_2_ or water activity at the Pt-ionomer interface from compression-induced densification. Lower cell voltages are observed for the edge-protected only MEA with exacerbated ohmic and mass transport losses and are consistent for replicate relief zone MEAs (Figure S3). The edge-protected only MEA experiences a GDL compression of 96% along the SG, indicating a high compression gradient that likely decreases to a 20% compression target at the MEA center. The porosity of the GDL in this case may be negatively impacted by limited O_2_ diffusion and the transfer of reactants and products in both the pore-structure and catalyst layer.^23,24^ Similar high frequency resistance (HFR) values indicate comparable interfacial contact and ionic resistance, suggesting performance differences are due to mass transport losses from GDL constriction.^25^ The edge-protected only MEA exhibits higher H_2_ crossover limiting current density measurements (*i*_H2_) at beginning of test (BOT), revealing that nonuniform overcompression can also drive PEM deformation and structural thinning at lands and intrusion into channels (Figure S4).^26^ Thus, relief zones can lower reactant crossover and enhance performance, enabling accurate and uniform GDL compression targets while mitigating PEM deformation.

Enhanced durability of the relief zone MEA was demonstrated during the combined chemical-mechanical accelerated stress test (AST) and evaluated using open circuit voltage (OCV) assessments and infrared (IR) thermography at the end of the test (EOT). Aggressive mechanical cycling (i.e., 100/0% RH at 30/45 s intervals) under an OCV hold condition assists in exposing imperfections in the MEA that arise from nonideal component integration either by fabrication and/or assembly techniques.^8,12^ The edge-protected only MEA approaches <0.9 V OCV at ∼250 h (Figure 3a). The corresponding IR thermography data at EOT show local heating along the top-edge of the MEA from the exothermic oxidation of H_2_ at exposed Pt sites, indicating through-plane perforation near the SG, whereas the relief zone MEAs approach <0.9 V OCV at 1.2 k and 1.4 k h for replicates 1 and 2, respectively, demonstrating a 6-fold enhancement (Figure 3c and 3e). The respective EOT IR thermography data for relief zone MEAs suggest H_2_ crossover by thinning, as indicated by broad, circular hot spots with lower relative temperatures located at or near flow-field outlets (Figure 3d and 3f).

**Figure 3:**
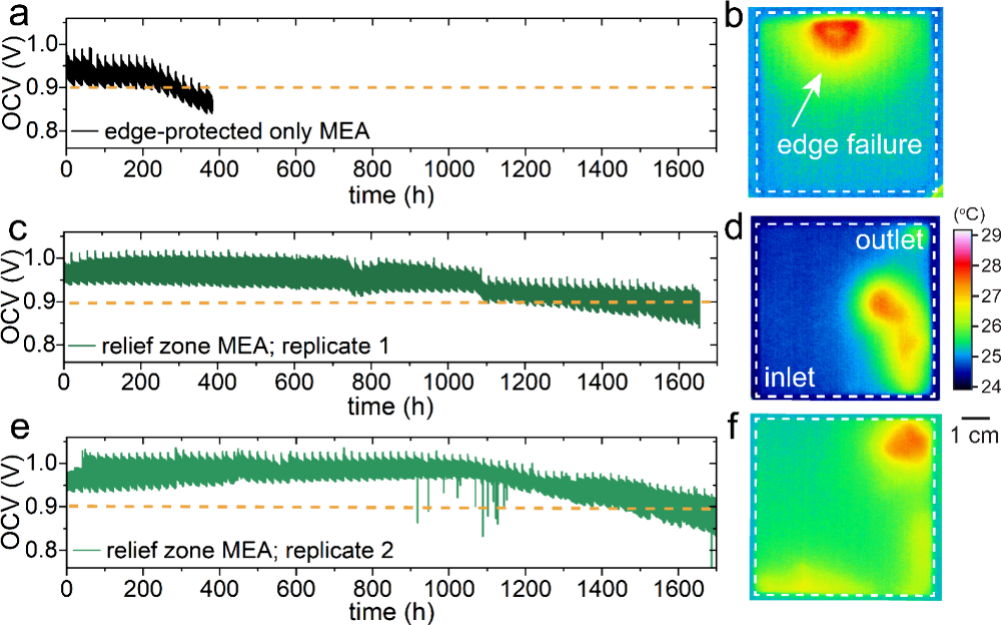
Open circuit voltage (OCV) profiles acquired from the combined chemical-mechanical accelerated stress test (AST) and the corresponding end of test (EOT) infrared (IR) thermography images are shown in (a, b) for the edge-protected only MEA; in (c, d) for the relief zone MEA, replicate 1; and in (e, f) for the relief zone MEA, replicate 2. All of the MEAs have the same orientation, as indicated in (d).

Cross-sectional, PEM thickness measurements of EOT samples acquired from failure, inlet, and outlet locations also indicate PEM thinning in relief zone MEAs (Figure S5). These data reveal thicknesses of 2.8 ± 0.38 μm and 11.7 ± 1.03 μm at PEM failure sites for the edge-protected only and relief zone MEAs, respectively. Pronounced loss of PEM material for the edge-protected MEA is demonstrated, whereas the relief zone MEA retains 78% of its initial thickness, corroborating the effectivity of the proposed method for mitigating mechanical degradation. Moreover, at the inlet and outlet locations, where pinhole failures are absent, relief zone PEMs exhibit retention of 72% and 89%, respectively, and with 1k h longer durability lifetime.

Interestingly, ICP-MS (Figure S6) analyses show that both MEAs exhibit higher Ce concentration at the outlets. At OCV, the PEM hydration state is dictated primarily by inlet humidification. Solvation-mediated transport of Ce and Fe toward outlets is driven by diffusional water gradients due to higher λ (H_2_O/SO_3_H) at the inlet.^27,28^ Higher mechanical stress and/or pressure gradients will also lower the λ for a PEM equilibrated with a specific water activity due to a reduction in free volume within ionomer water domains.^29^ Consequently, the edge-protected only MEA experiences an amplified hydraulic potential gradient from the center of the active area toward the inactive regions beneath the CG. This effect manifests as reduced Ce retention and a higher transport of Ce from active to inactive areas, as measured by XRF (Figure S7), whereas the relief zone architecture minimizes compression at the active area boundary, maintaining a higher local water content and a more uniform hydration profile. By reducing the magnitude of the water flux driving force, this design suppresses the convective displacement of scavengers, resulting in the higher Ce retention within the active area and lowering residual Fe content. To further resolve underlying PEM failure modes, OCV transient analyses were used to leverage deviations from predicted voltage values under defined operating conditions.

Deviations from thermodynamically derived OCV predictions under specific operating conditions can be used to monitor irreversible degradation events. Figures 4a and 4b show the deconvoluted OCV transients for low and high RH conditions. Nernstian behavior and kinetic effects elicit higher dry OCV responses due to greater reactant partial pressures at low RH. Herein, nonrecoverable losses are attributed to PEM degradation, as demonstrated through declines in dry OCV as losses from H_2_ crossover increase.^30^ Critical failures in Figure 4a and 4b (i.e., dashed boxes) are evident where similar OCVs occur under dry and wet conditions, resulting in a ΔOCV of 0 mV (i.e., wet OCV subtracted from dry OCV).^8,9^ A reversal of the dry and wet OCV responses (i.e., wet OCV ≫ dry OCV) can indicate PEM perforation, since H_2_ crossover is attenuated by water uptake and swelling and higher crossover losses occur in the dry state. Interestingly, the relief zone MEAs do not exhibit this reversal behavior (Figure 4b) but rather scattering behavior evidently from thinning rather than perforation. Convergence of dry and wet OCV responses where ΔOCV remains near 0 mV occur for 200–300 h. Zoomed-in transient plots of this behavior are provided in Figures S8 and S9. Use of relief zones in combination with reinforcement layers suggests that through-plane perforations from mechanical stresses are mitigated such that thinning becomes the dominant failure mode.

**Figure 4:**
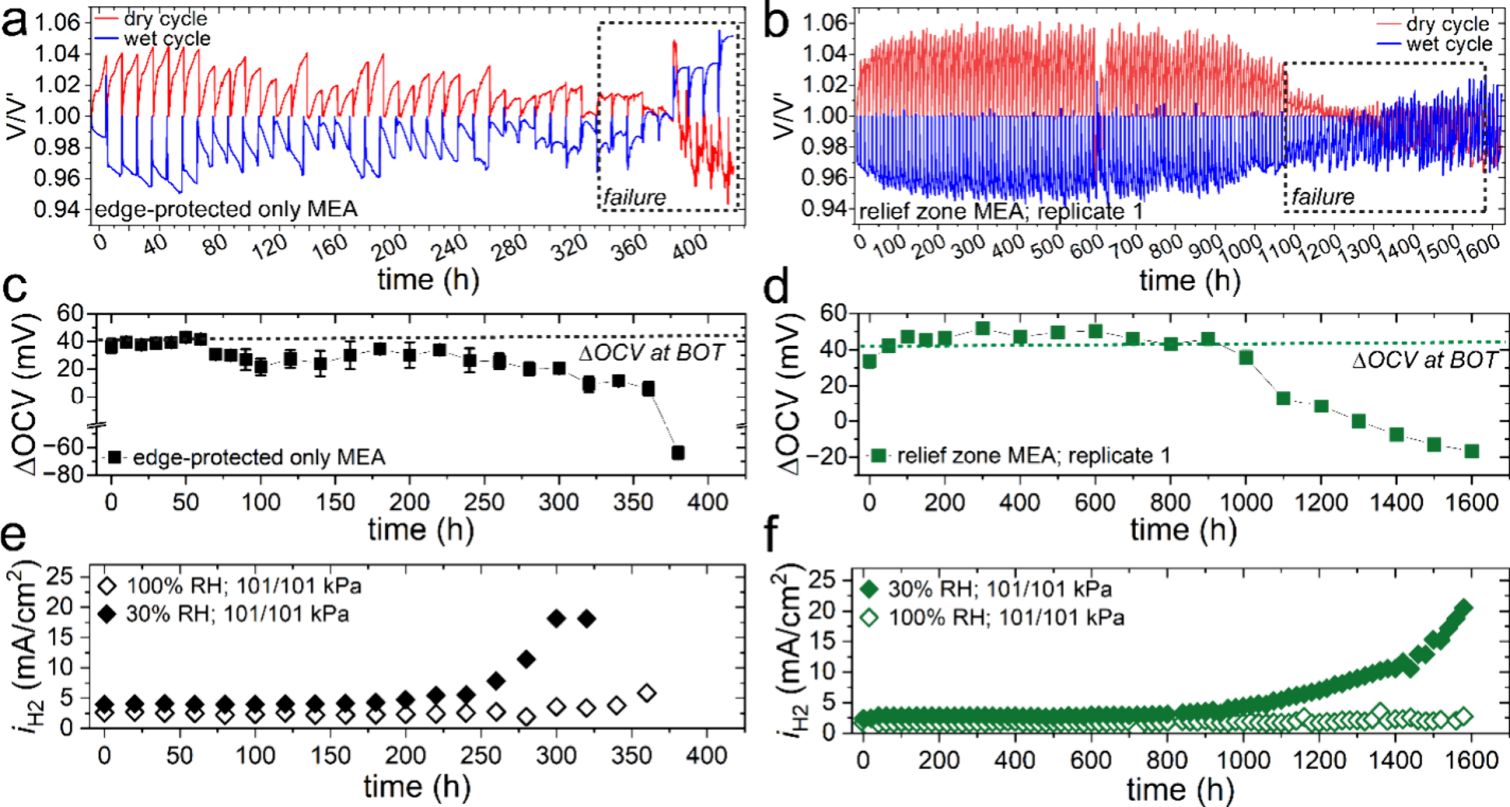
OCV transients for the (a) edge-protected only MEA and the (b) relief zone MEA, replicate 1, where a single voltage cycle was extracted 500 s prior to the end of each 10 h interval and normalized to the initial voltage (V′). The dashed boxes in (a) and (b) highlight the region of failure. The ΔOCV for selected time points during the AST for the (c) edge-protected only MEA and the (d) relief zone MEA, replicate 1, calculated from the last voltage value at the end of each cycle. The H_2_ crossover limiting current density measurements (*i*_H2_) acquired at 20 h intervals for the (e) edge-protected only MEA and the (f) relief zone MEA, replicate 1.

Figures 4c and 4d show the ΔOCV values at selected intervals. The ΔOCV at BOT was determined as 41 mV (dashed line), defined by the Nernst potential corrected for H_2_ crossover and cathodic kinetic losses at both low and high RH.^9^ The edge-protected MEA in Figure 4c exhibits a decrease in ΔOCV after 70 h, while increases to *i*_H2_ are not apparent until 200 h at 30% RH (Figure 4e). In contrast, decreases in ΔOCV track with increasing *i*_H2_ at 30% RH for the relief zone MEA (Figure 4d and 4f). Higher and/or nonuniform effective PEM hydration from overcompression of the GDL can result in slowed water transport and, therefore, obfuscated *i*_H2_ values. Similar divergences between ΔOCV declines and crossover were previously encountered for cracked electrodes with increased and nonuniform water retention.^8^ Thus, relying on *i*_H2_ alone does not fully capture the PEM degradation mechanisms. Similarly, the relief zone MEA exhibits little to no increase for *i*_H2_ at 100% RH, as this measurement does not effectively capture the thinning behavior. To further elucidate such failure modes, a series of *i*_H2_ measurements were performed to separate diffusive (*i*_H2_ *diffusive*) and convective (*i*_H2_ *convective*) crossover contributions to distinguish between thinning and through-plane perforations.

Figures 5a and 5b show the *i*_H2_ versus H_2_ mole fraction (Χ_H2_ = 0.1, 0.3, 0.6, and 1.0) for symmetric (ΔP = 0 kPa) and differential backpressure (ΔP = 30 kPa) conditions for the edge-protected only and relief zone MEA, respectively. In brief, slopes for ΔP = 0 kPa represent *i*_H2_ *diffusive*, while slopes for ΔP = 30 kPa include both *i*_H2_ *diffusive* and *i*_H2_ *convective* terms, and the y-intercept at Χ_H2_ = 0 represents the contribution from shorting current.^27^ At BOT, the *i*_H2_ *diffusive* and *i*_H2_ *convective* terms are similar in magnitude for the edge-protected only (i.e., 4.16 mA/cm^2^ and 60.2 μA/cm^2^) and the relief zone MEA (i.e., 4.09 mA/cm^2^ and 35.7 μA/cm^2^). Excessive compression and a high pressure gradient caused early failures near the SG frame in edge-protected only MEAs, as demonstrated by an increase to 21.9 mA/cm^2^ for *i*_H2_ *convective* at EOT (Figure 5a). In contrast, the relief zone MEA exhibits a modest increase where *i*_H2_ *convective* = 1.82 mA/cm^2^ (Figure 5b). The time-resolved breakdown in Figure 5c shows an increase to *i*_H2_ *diffusive* and minimal *i*_H2_ *convective* at EOT for the relief zone MEA. In short, the relatively low *i*_H2_ *convective* contribution further suggests that the reinforced PEMs with relief zones exhibit thinning as a failure mode even when subjected to aggressive mechanical cycling during the AST. In contrast, excess and nonuniform compression in edge-protected only MEAs result in early failures which obscure assessments toward intrinsic PEM material properties.

**Figure 5:**
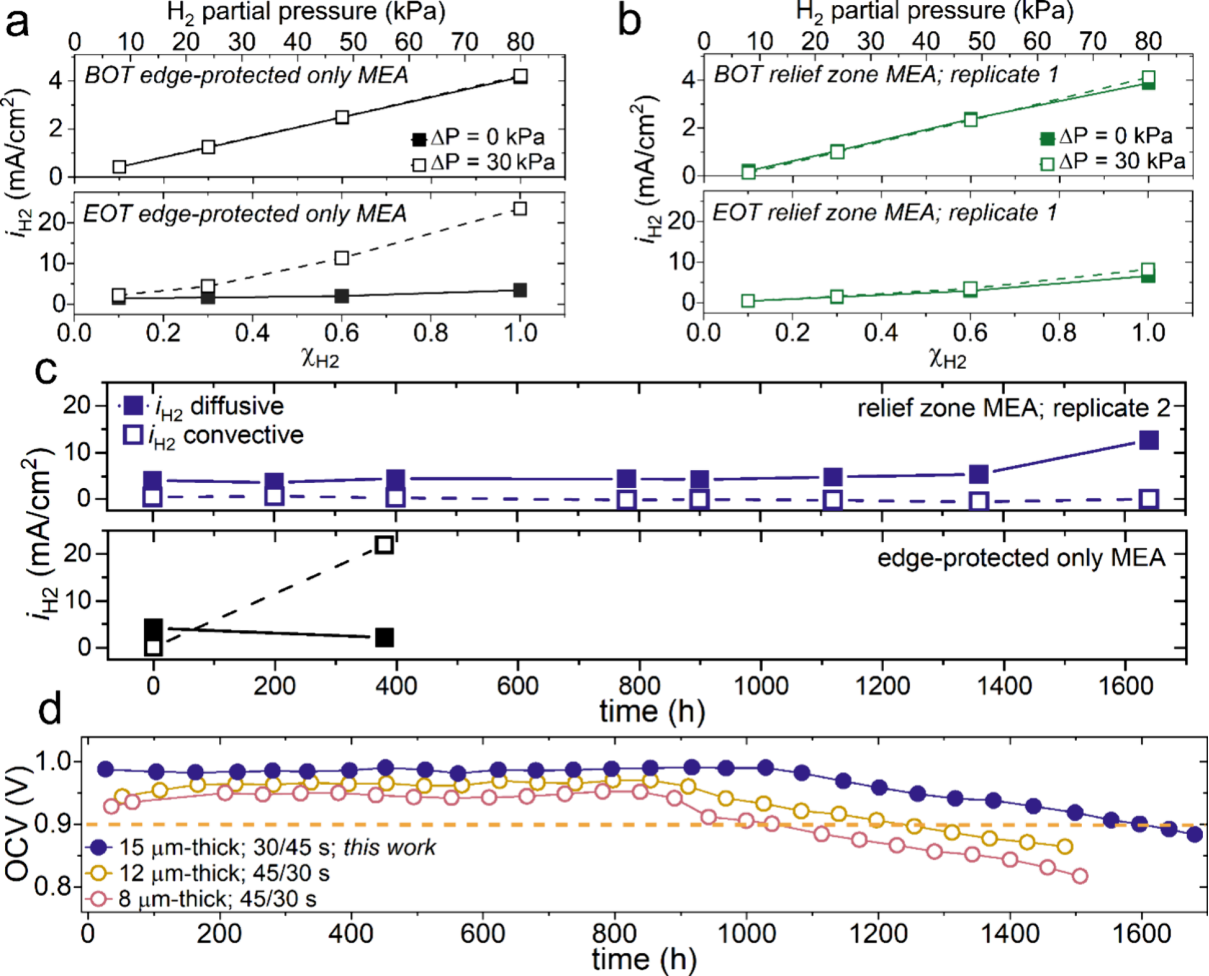
*i*_H2_ versus H_2_ mole fractions (Χ_H2_) at 100% RH under Δ*P* = 0 and Δ*P* = 30 kPa for the (a) relief zone MEA, replicate 1 and the (b) edge-protected only MEA at beginning of test (BOT) and EOT. The (c) time-resolved *i*_H2_ convective and diffusive contributions for the edge-protected only and relief zone, replicate 2 MEAs. The (d) OCV performance data evaluated for other ultrathin, reinforced PEMs compared to our work using the combined chemical-mechanical AST.^13^ It should be noted that this work was performed using a more aggressive, longer dry cycle.

Distinguishing between intrinsic material properties and integration-driven failure modes is critical to directing future research endeavors and yielding a proper assessment of the state of technology for subsequent scale-up. While thickness-independent durability for ultrathin reinforced PEMs has been reported for combined chemical mechanical ASTs, the results were ultimately inconclusive due to early mechanical failures from stress concentrations along the frames. Figure 5d compares the durability of relief zone MEAs against 8 and 12 μm-thick PEMs probed under less aggressive conditions (i.e., shorter dry cycle, 45/30 s wet/dry).^13^ While this comparison provides a qualitative understanding, it underscores the importance of optimizing device-level integration prior to material assessment and showcases relief zones as an effective strategy for reducing local stress concentrations near electrode edges inherent to traditional methods of edge-protection.

To properly assess material properties within small scale devices, we move past limitations in single-cell assembly techniques toward robust integration of ultrathin PEM materials. As material properties such as hydration, swelling, and thickness change, revisiting cell-level engineering to understand the boundary conditions is critical for proper material assessments. Herein, we show that relief zones can be applied to improve the signal-to-noise ratio of material properties over cell-level engineering to not only yield accurate assessments of material limitations but also properly define failure modes for subsequent material-based mitigation strategies. Root causes of early mechanical failures were identified and mitigated using a stress relief zone strategy, demonstrating a 6-fold improvement in AST lifetime. Analyses of scavenger retentions in post-mortem MEAs highlighted the critical role of structural architecture in governing these distributions and suggest further investigation into the coupled effects of mechanical and chemical degradation. Linkage of absolute pressures along lands and the resulting diffusion media compression could guide new metrics to mitigate structural impacts. These findings could assist in an improved translation of representative durability characterizations from single-cell- to stack-level platforms.

## Supplementary Material



## Notes

The Supporting Information includes the experimental methods, optical, scanning electron microscopy, pressure film images, polarization, H_2_ crossover limiting current density and membrane thickness measurements, the ICP-MS and XRF results for the MEAs at end of test, and the open circuit voltage transients. (PDF)

## References

[1] Gittleman C. S., Jia H., De Castro E. S., Chisholm C. R. I., Kim Y. S. (2021). Proton Conductors
for Heavy-Duty Vehicle Fuel Cells. Joule.

[2] Kienitz B., Kolde J., Priester S., Baczkowski C., Crum M. (2011). Ultra-Thin Reinforced Ionomer Membranes to Meet Next Generation Fuel
Cell Targets. ECS Trans..

[3] Luo X., Lau G., Tesfaye M., Arthurs C. R., Cordova I., Wang C., Yandrasits M., Kusoglu A. (2021). Thickness Dependence of Proton-Exchange-Membrane
Properties. J. Electrochem. Soc..

[4] Cullen D. A., Neyerlin K. C., Ahluwalia R. K., Mukundan R., More K. L., Borup R. L., Weber A. Z., Myers D. J., Kusoglu A. (2021). New Roads
and Challenges for Fuel Cells in Heavy-Duty Transportation. Nature Energy.

[5] Gittleman C. S., Kongkanand A., Masten D., Gu W. (2019). Materials
Research
and Development Focus Areas for Low Cost Automotive Proton-Exchange
Membrane Fuel Cells. Current Opinion in Electrochemistry.

[6] Kienitz B. (2021). Optimizing
Polymer Electrolyte Membrane Thickness to Maximize Fuel Cell Vehicle
Range. Int. J. Hydrogen Energy.

[7] Shi S., Weber A. Z., Kusoglu A. (2016). Structure/Property Relationship of
Nafion XL Composite Membranes. J. Membr. Sci..

[8] Taylor A. K., Baez-Cotto C., Hu L., Smith C., Rodriguez-Nazario A., Young J. L., Mauger S., Neyerlin K. C. (2025). The Influence of
Electrode Crack Dimensions on the Durability of Polymer Electrolyte
Membrane Fuel Cells. J. Power Sources.

[9] Taylor A. K., Smith C., Neyerlin K. C. (2023). Mitigation
and Diagnosis of Pin-Hole
Formation in Polymer Electrolyte Membrane Fuel Cells. J. Power Sources.

[10] Wang M., Medina S., Ochoa-Lozano J., Mauger S., Pylypenko S., Ulsh M., Bender G. (2021). Visualization,
Understanding, and
Mitigation of Process-Induced-Membrane Irregularities in Gas Diffusion
Electrode-Based Polymer Electrolyte Membrane Fuel Cells. Int. J. Hydrogen Energy.

[11] Qiu D., Peng L., Liang P., Yi P., Lai X. (2018). Mechanical
Degradation of Proton Exchange Membrane Along the MEA Frame in Proton
Exchange Membrane Fuel Cells. Energy.

[12] Wang M., Taylor A. K., Ochoa-Lozano J., Medina S., Pfeilsticker J. R., Mauger S. A., Pylypenko S., Ulsh M., Bender G. (2025). The Impact
of Hot-Press Conditions on the Durability of Polymer Electrolyte Membrane
Fuel Cells. Int. J. Hydrogen Energy.

[13] Yao Z., Zhou F., Tu C., Tan J., Pan M. (2024). Decay Behaviour
of Ultrathin Reinforced Membranes in PEMFCs Subjected to the Combination
of Mechanical/Chemical Accelerated Stress Testing. Int. J. Hydrogen Energy.

[14] James, R. L. ; Valentine, S. D. ; Liestra, J. Manufacture or Membrane Electrode Assembly with Edge Protection for PEM Fuel Cells. US Patent US8,470,497: 2006.

[15] Leistra, J. ; James, R. L. ; Dobulis, D. Controlled Electrode Overlap Architecture for Improved MEA Durability. U.S. Patent US7,955,750: 2011.

[16] Yang D., Tan Y., Li B., Ming P., Xiao Q., Zhang C. (2022). A Review of
the Transition Region of Membrane Electrode Assembly of Proton Exchange
Membrane Fuel Cells: Design, Degradation, and Mitigation. Membranes.

[17] Kink J., Suermann M., Ise M., Bensmann B., Junker P., Hanke-Rauschenbach R. (2024). Reinforcing Membranes with Subgaskets
in Proton Exchange
Membrane Water Electrolysis: A Model-Based Analysis. J. Power Sources.

[18] Wang M., Rome G., Phillips A., Ulsh M., Bender G. (2019). Effective
Electrode Edge Protection for Proton Exchange Membrane Fuel Cell Drive
Cycle Operation. ECS Trans.

[19] Sompalli, B. ; Gasteiger, H. A. ; Litteer, B. A. ; Yan, S. G. Edge-Protected Catalyst-Coated Diffusion Media and Membrane Electrode Assemblies. Patents US8,007,949: 2011.

[20] Sassin M., Garsany Y., Gould B., Swider-Lyons K. (2016). Impact of
Compressive Stress on MEA Pore Structure and Its Consequence on PEMFC
Performance. J. Electrochem. Soc..

[21] Lee D. U., Joensen B., Jenny J., Ehlinger V. M., Lee S.-W., Abiose K., Xu Y., Sarkar A., Lin T. Y., Hahn C. (2023). Controlling Mass Transport
in Direct Carbon Dioxide Zero-Gap Electrolyzers
Via Cell Compression. ACS Sustainable Chem.
Eng..

[22] García-Salaberri P. A., van Eijk L., Bangay W., Ferner K. J., Ha M. H., Moore M., Perea I., Kusoglu A., Secanell M., Das P. K. (2025). Materials Engineering
for High Performance and Durability
Proton Exchange Membrane Water Electrolyzers. ACS Appl. Energy Mater..

[23] Zenyuk I. V., Parkinson D. Y., Connolly L. G., Weber A. Z. (2016). Gas-Diffusion-Layer
Structural Properties under Compression Via X-Ray Tomography. J. Power Sources.

[24] Atkinson
III R. W., Garsany Y., Gould B. D., Swider-Lyons K. E., Zenyuk I. V. (2018). The Role of Compressive Stress on Gas Diffusion Media
Morphology and Fuel Cell Performance. ACS Appl.
Energy Mater..

[25] Simon C., Hasché F., Gasteiger H. A. (2017). Influence of the Gas Diffusion Layer
Compression on the Oxygen Transport in Pem Fuel Cells at High Water
Saturation Levels. J. Electrochem. Soc..

[26] Kulkarni N., Kok M. D., Jervis R., Iacoviello F., Meyer Q., Shearing P. R., Brett D. J. (2019). The Effect
of Non-Uniform
Compression and Flow-Field Arrangements on Membrane Electrode Assemblies-X-Ray
Computed Tomography Characterisation and Effective Parameter Determination. J. Power Sources.

[27] Lai Y. H., Rahmoeller K. M., Hurst J. H., Kukreja R. S., Atwan M., Maslyn A. J., Gittleman C. S. (2018). Accelerated Stress Testing of Fuel
Cell Membranes Subjected to Combined Mechanical/Chemical Stressors
and Cerium Migration. J. Electrochem. Soc..

[28] Baker A. M., Mukundan R., Spernjak D., Judge E. J., Advani S. G., Prasad A. K., Borup R. L. (2016). Cerium Migration During PEM Fuel
Cell Accelerated Stress Testing. J. Electrochem.
Soc..

[29] Kusoglu A., Kienitz B. L., Weber A. Z. (2011). Understanding the Effects of Compression
and Constraints on Water Uptake of Fuel-Cell Membranes. J. Electrochem. Soc..

[30] Kundu S., Fowler M., Simon L. C., Abouatallah R. (2008). Reversible
and Irreversible Degradation in Fuel Cells During Open Circuit Voltage
Durability Testing. J. Power Sources.

