# Supplementary material for: Relief Zones Enhance the Durability of Ultrathin Membranes in Electrochemical Conversion Devices
Source: ACS Appl Energy Mater. 2026 Feb 4;9(4):2050–6. doi: 10.1021/acsaem.5c03843 (PMC12933513; doi:10.1021/acsaem.5c03843)
Supplement: Supplementary file 1 [file ae5c03843_si_001.pdf]

Supporting Information

“Relief Zones Enhance the Durability of Ultra-Thin Membranes in Electrochemical Conversion Devices”

Audrey K. Taylor [a], Megan McVeigh [a], Catherine Weiss [b], and Kenneth C. Neyerlin [a,]*

[a] Chemistry and Nanoscience Center, National Laboratory of the Rockies, Golden, CO, 80401, USA

[b] Energy Technologies Area, Lawrence Berkely National Laboratory, Berkely, CA, 94720, USA

[*]Corresponding Author: **Kenneth.neyerlin@nlr.gov*

1. **Experimental Methods**

1.1 *Materials and Reagents*

Reinforced and chemically stabilized PEMs (NC700, 15 μm-thick, The Chemours Company FC, LLC) were used without any pre-treatment. The catalyst for all of the MEAs was a platinum on high surface area carbon (Pt/C, TEC10E50E, 46.7 wt.%, Tanaka Kikinzoku Kyogo), and the ionomer (D2020, 20 wt.%) with an equivalent weight (EW) of 920 was obtained from Ion Power. Preparation of the catalyst ink included 1-propanol (n-PA, HPLC grade, Sigma Aldrich) and high-purity deionized water (DI, 18 MΩ•cm, Millipore, Mill-Q Advantage A10 filtration system) solvents. All of the gas diffusion media, Freudenberg (H23C8, Freudenberg Performance Materials SE and CO KG, with a nominal thickness of 264 ± 2 μm) was calendered prior to use as per established methods.[1] Gylon® and poly(imide) films were acquired from CS Hyde and McMaster Carr, respectively and used in the calendering process. The edge protection (EP) material consisted of thin poly(imide) sheets (7 μm-thick, CS Hyde) custom cut to specify a total cell area of 50 cm^2. The hard-stop, compression gaskets (CG) were poly(tetrafluoroethylene) (PTFE, McMaster Carr) and custom-cut to provide an oversized active area of 50 cm^2 on the quad serpentine, flow-fields (graphite blocks, Poco®, Fuel Cell Technologies). Fuel cell testing included the use of hydrogen (H_2), nitrogen (N_2), and oxygen (O_2) with gas purities of 99.95, 99.999, and 99.994%, respectively.

1.2 *Fabrication of the Catalyst Coated Membranes*

Catalyst coated membranes (CCMs) were prepared by spray deposition using a Sonotek Exacta Coat system (25 kHz Accumist nozzle) to achieve target loadings of 0.1 and 0.2 mg/cm^2

for the Pt/C anodes and cathodes, respectively. The catalyst ink was prepared directly prior to spray deposition. The solid Pt/C was transferred to a clean vial followed by the addition of DI, n-PA, and D2020 to obtain a final dispersion of 0.2 wt.% Pt. The DI to n-PA solvent ratio was 62/38 wt.% with an ionomer to carbon ratio (I/C) of 0.9. The contents of the vial was horn sonicated (SFX250, 250 W, tip diameter: ½, Branson Misonix) for 10 s followed by an ice bath sonication for 20 min (FS30, 100 W, Fisher Scientific). The ink was transferred to a syringe and pumped at a rate of 0.3 mL/min during the spray deposition. Porous PTFE was placed onto a heated vacuum table (80 ^{o}C, 15 psi) to provide a smooth, clean surface for the pre-cut sections of the PEM to adhere to on the table. Custom cut spray masks of PTFE-coated fiberglass were placed over-top the sections of PEM to define the spray area and provide an improved vacuum. The catalyst loadings were confirmed using a X-ray fluorescent spectrometer (XRF, Fischer scope XDV-SDD, 50 kV, 50 W X-ray source) after the CCM fabrication.

1.3 *Fabrication of the Stress Relief Zones*

Laser ablation of the diffusion media was performed using a ULTRA R5000, Universal Laser system to create stress relief zones. The stress relief zones were processed using a top-down raster technique with specified dimensions of 2 mm in width and a depth of 103 ± 7 μm along all four edges of the diffusion media. This ablation was performed on the top-most layer composed of carbon fibers leaving behind an intact microporous layer (MPL) beneath the relief zone. The relief zone in the diffusion media ultimately alleviated strain concentration and, therefore, a more uniform compression across the active area of the MEA was achieved toward robust durability characterizations of the ultra-thin PEMs.

1.4 *Assembly of the Polymer Electrolyte Membrane Fuel Cell*

The diffusion media was calendered prior to use with a downward force of 25 kg/cm^2 using a Carver press (model #3912). This process mitigates the occurrence of membrane irregularities encountered in the subsequent assembly steps.[2, 3] To further ensure that the CCMs were irregularity-free, infrared thermography (IR) was performed prior to beginning of test (BOT).[4] All of the MEAs were fabricated using sub-gaskets (SGs) as per established methods.[5, 6] The SG overlap with the diffusion media was 1.85 mm in width along all four, which defined the 50 cm^2 active area. The compression gaskets (CGs) were 0.15 to 0.18 mm in thickness to target a diffusion media compression of 20%. This hard-stop sealing strategy considers a 6% compressibility of the CGs. These components (i.e., CCM, SG, and CG) were assembled between the graphite flow-fields with close attention to their alignment and guided using alignment pins. A single CG was placed onto the flow-field followed by placement of pre-cut, diffusion media within the CG opening. The SG material then followed, ensuring an adequate planar overlap was achieved on all four sides followed by the central CCM layer. Assembly was completed after the symmetrical placement of similar components making up the cathode side. The end plate of the hardware was fastened using Belleville compression washers. Every third bolt was tightened at 2.25 N-m followed by 4.5 N-m. The assembled hardware underwent a leak test to ensure adequate sealing by closing the outlet ports on the hardware and filling the cell with 2 bar of air. A drop in this pressure hold measurement indicated a leak. Any leaks were addressed by re-assembling the hardware in an effort to improve the alignment of the MEA components to obtain a leak-free hardware.

1.5 *Electrochemical Testing*

The fuel cell test station was a customized Hydrogenics station equipped with LabView programming software and a Gamry 3000 potentiostat. Gradual heating of the cell to 80 ^{o}C was performed using H_2/N_2 (anode/cathode) in co-flow with a relative humidity (RH) of 100% and absolute backpressure of 150/150 kPa. An initial AC impedance measurement was acquired at a potential of 0.45 V in H_2/N_2 with a flow of 0.3/0.7 slpm. This measurement was obtained to inform an appropriate high frequency resistance (HFR) value for use in subsequent performance measurements. For example, single frequency HFR values were recorded during current measurements to inform the ohmic resistance of the cell.

Break-in and a voltage recovery protocol was performed at BOT.[7] The cell break-in consisted of a series of voltage holds in H_2/air, 80 ^{o}C, 100% RH, and 150/150 kPa as described in past work.[8] The voltage recovery protocol included low temperature and high RH voltage cycling in H_2/O_2. Polarization measurements were also performed between each round of voltage recovery at 100% RH and 150/150 kPa in H_2/air.

1.6 *Durability Testing and Diagnostics*

The accelerated stress test (AST) was performed according to the protocol described in the Fuel Cell Technical Roadmap.[9] This combined chemical mechanical AST included an open circuit voltage (OCV) hold at 90 ^{o}C in H_2/air at 2/2 slpm with a backpressure of 101/101 kPa. Mechanical stressors were simultaneously induced by cycling the anode and cathode reactant gas humidities between wet and dry conditions specified by a 30/45 s interval. It should be noted that a longer dry period (45 s) relative to the wet flow (30 s) condition was used for the durability tests. A series of H_2 crossover limiting current density measurements (i_{H2}) were performed after AST segments of

20 h. The i_{H2} are performed by purging the cell with H_2 at the anode and N_2 at the cathode. Subsequently a 0.4 V potential is applied across the cell to oxidize H_2 that crosses over to the cathodic, working electrode. Low and high RH conditions (i.e., 30 and 100% RH) were used to measure i_{H2} at 101/101, 150/150, and 150/101 kPa and with a cell temperature of 90 ^{o}C and with a 30 min equilibration period for low RH. Additionally, a H_2 crossover breakdown was used to distinguish the diffusive (i_{H2} *diffusive*) and convective (i_{H2} *convective*) terms performed using methods described by Lai *et al.*[10] For this breakdown, a series of mole fractions of H_2 (X_{H2} = 1.0, 0.6, 0.3, 0.1), diluted with N_2, were measured at two backpressure conditions (i.e., 150/120 and 150/150 kPa for ΔP = 30 kPa and ΔP = 0 kPa, respectively) at 90 ^{o}C and 100% RH. A linear regression analysis was used to assign the slopes to the i_{H2} *diffusive* and i_{H2} *convective* terms as described in the prior work. The i_{H2} breakdown was measured at BOT and EOT for the edge-protected only and relief zone MEA; replicate 1. Additional time-resolved i_{H2} breakdown were provided only for relief zone MEA; replicate 2.

1.7 *Contact Pressure Distribution Analyses*

Pressure sensitive film was used to measure the contact pressure distribution across the areas of the edge-protected and relief zone MEAs within single-cell hardware. These characterizations were performed within the cell hardware which was assembled under similar conditions that were representative of electrochemical testing. Pre-cut Fujifilm Prescale LLW (0.5 – 2.5 MPa) films were placed between the flow-fields using the alignment pinholes and along with the MEA. This process effectively captured the variation in pressure experienced in an operating cell. These resultant films were analyzed using the Fujifilm Pressure Sensor Scanner System and FPD-8010E software.

1.8 *End of Test Characterization of the Membrane Electrode Assemblies*

1.8.1 *Scanning Electron Microscopy*

Cross-section images of the post-mortem CCMs were acquired using a scanning electron microscopy (SEM, FEI Quanta FEG 250) to inspect the end of test (EOT) polymer electrolyte membrane (PEM) thickness. Sample preparation for SEM included removal of the gas diffusion media from the CCMs. Small sections of the CCM from the inlet, outlet, and middle of the MEA were cut out for inspection by SEM. The CCM cutouts were submerged in liquid N_2 for 5 min and fractured while submerged. The cross-sectional samples were mounted in the specimen chamber under high vacuum ($< 2 \times 10^{-5}$ Torr). The energy level of the beam was set to 5 kV to minimize beam damage to the ionomer.

1.8.2 *Infrared Thermography*

The infrared (IR) thermography was used to spatially detect through-plane cracks or pin-holes in the PEM after EOT. It should be noted that IR thermography was also used to inspect each MEA prior to hardware assembly and electrochemical testing to ensure a defect-free, intact PEM. The MEAs were positioned at the opening of an *ex situ* cell. The cell was purged with N_2 prior to flowing the N_2 diluted H_2. A Jenoptik Vario-CAM was used to capture IR images of the MEA.

1.8.3 *ICP-MS*

The iron (Fe) and cerium (Ce) quantifications were measured using Agilent 7900 ICP-MS. Small areas (approximately 2 x 2 cm^2) from the inlet, outlet, and center were cut out of the CCMs. The samples were soaked in 20 mL of 5% ICP-MS grade nitric acid (HNO_3) for 2 weeks, twice.

The HNO_3 was filtered using 0.2 μm filter syringe prior to injection to ensure no Pt/C particles enter the ICP-MS.

$$\text{relief zone depth} \geq (\delta_{GDL} \times \% \text{ compression})$$

Equation S1. A target relief zone depth was obtained using the known thickness of the gas diffusion layer (δ_{GDL}) and the pre-determined GDL compression target.

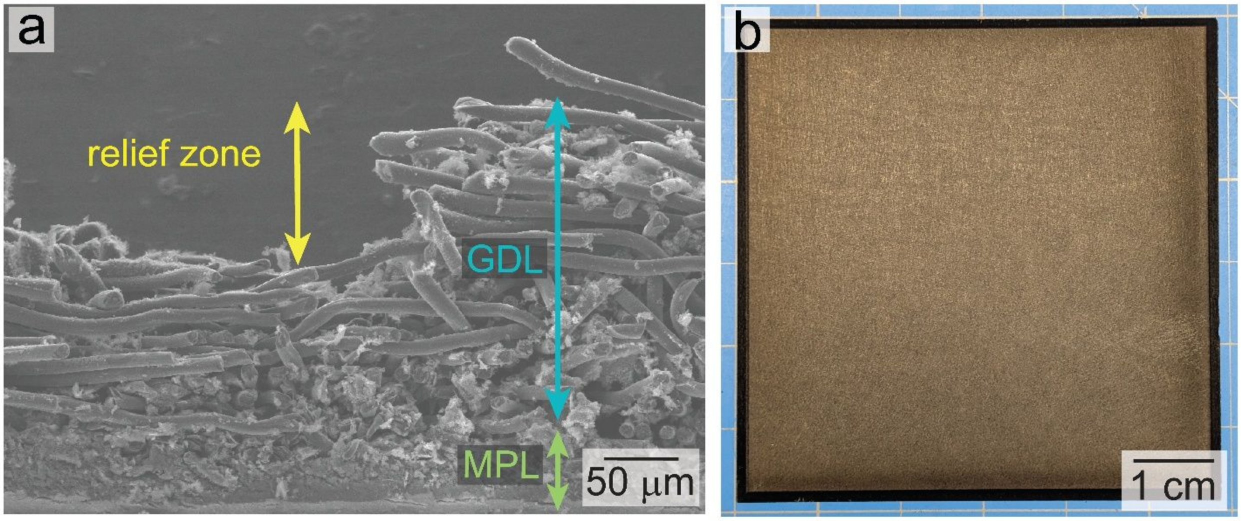

Figure S1. Representative scanning electron microscopy (SEM) image of the (a) relief zone cross-section showing the ablation depth. The (b) top-down, optical image of the GDL with relief zones along all four edges.

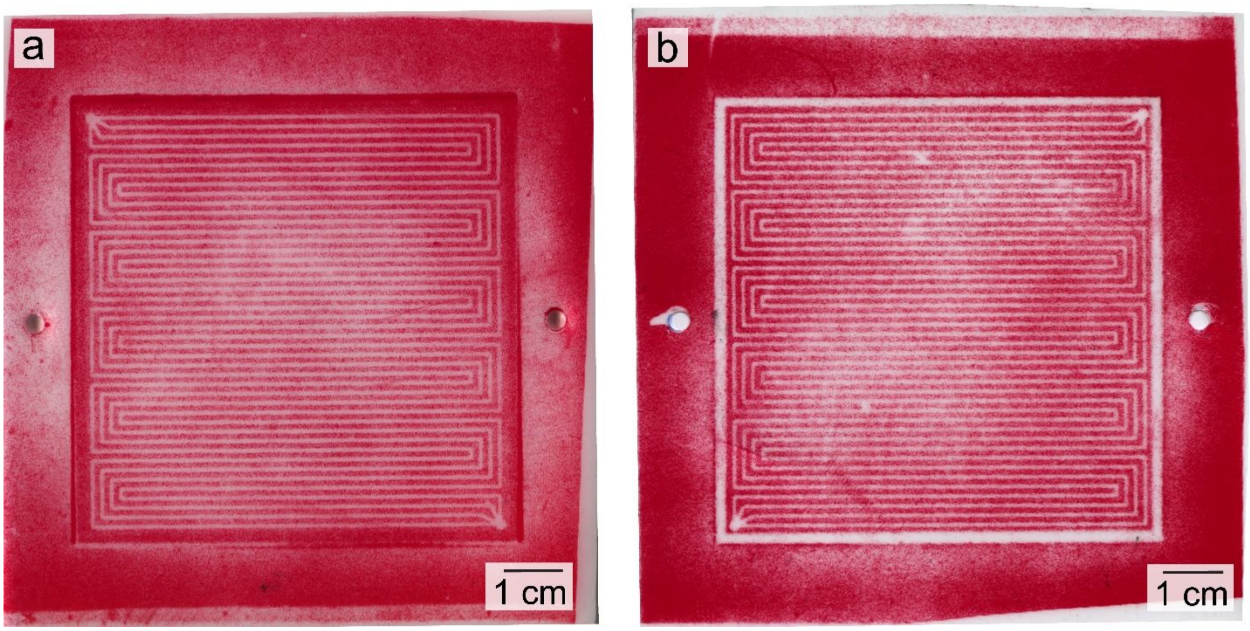

Figure S2. The compression film measurements of the (a) edge-protected and the (b) relief zone membrane electrode assemblies (MEAs) using 7 μm-thick poly(imide) sub-gaskets.

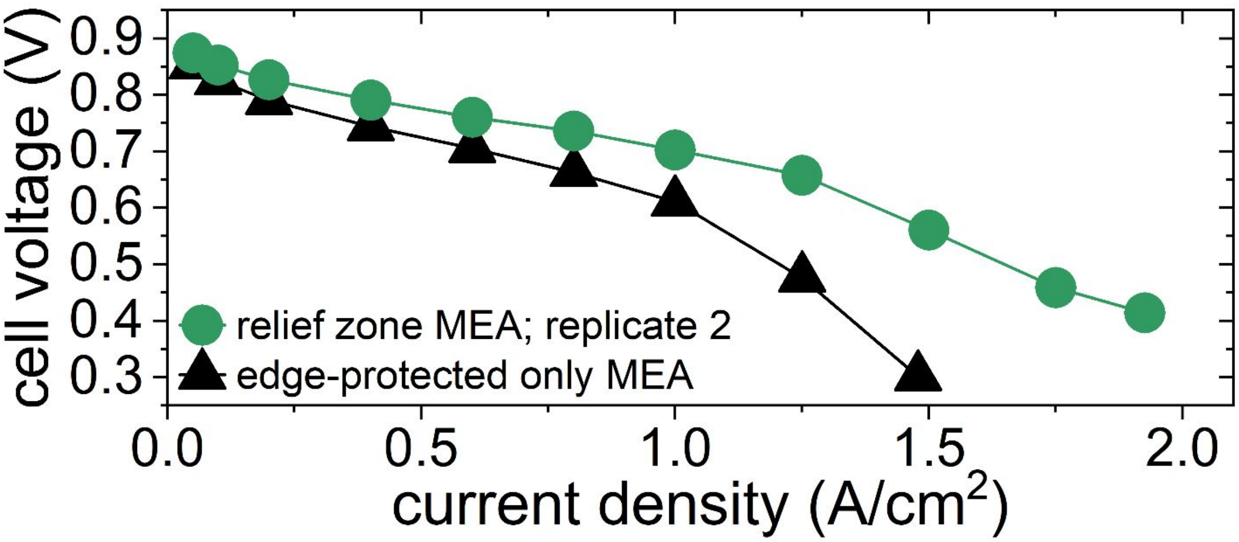

Figure S3. The polarization curves acquired in H_2/air at 80 ^oC, 100% RH, and 150/150 kPa for the relief zone MEA; replicate 2 and the edge-protection only MEA.

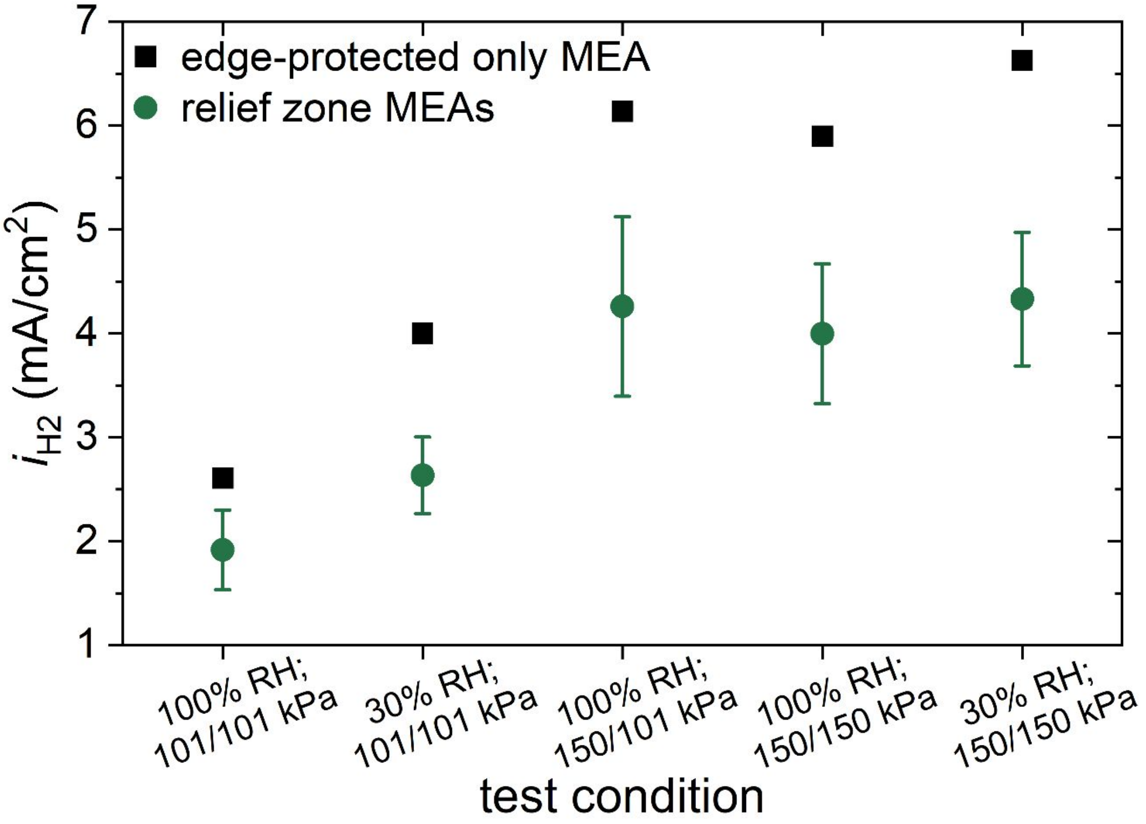

Figure S4. The beginning of test (BOT) H_2 crossover limiting current density measurements (i_{H2}) for a series of test conditions acquired at 30 and 100% RH both at 101/101 and 150/150 kPa as well as 100% RH at a differential backpressure condition of 150/101 kPa. All of the data were acquired at a cell temperature of 90 ^{o}C for a NC700 PEM (15 μm-thick).

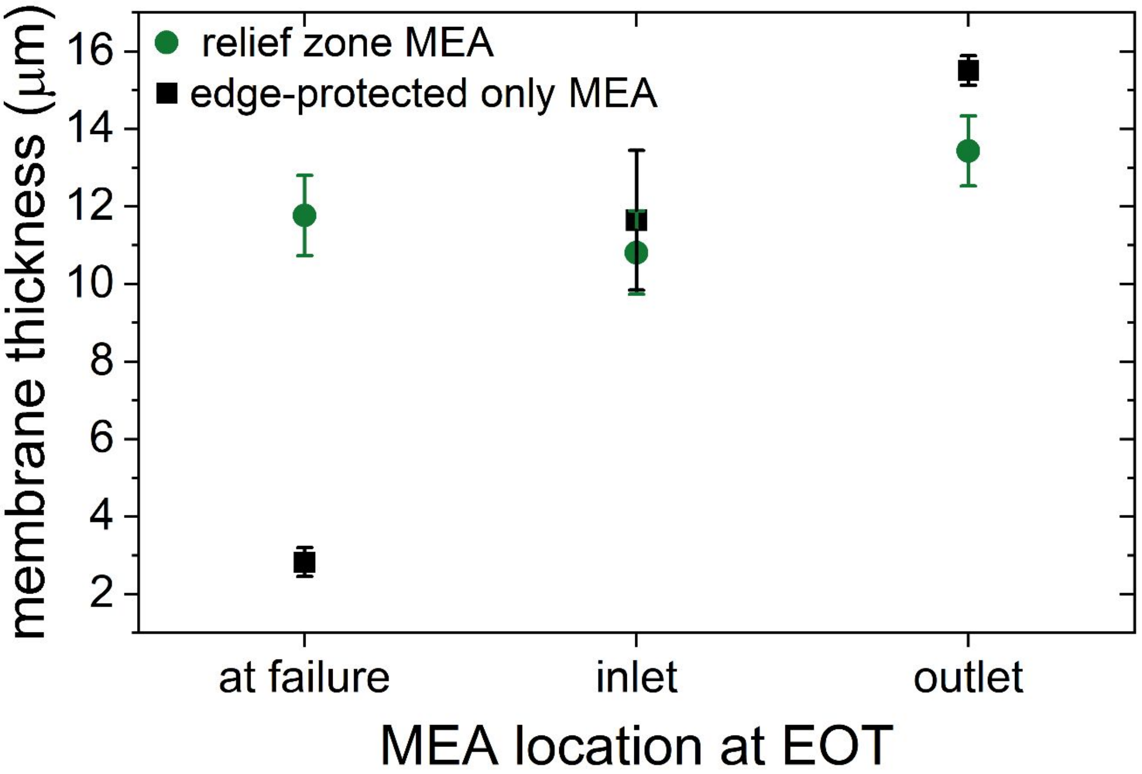

Figure S5. Membrane thickness measurements acquired from cross-sectional scanning electron microscopy (SEM) images at failure, inlet, and outlet locations from the MEA at end of test (EOT). The data for the relief zone MEA were acquired from replicate 2.

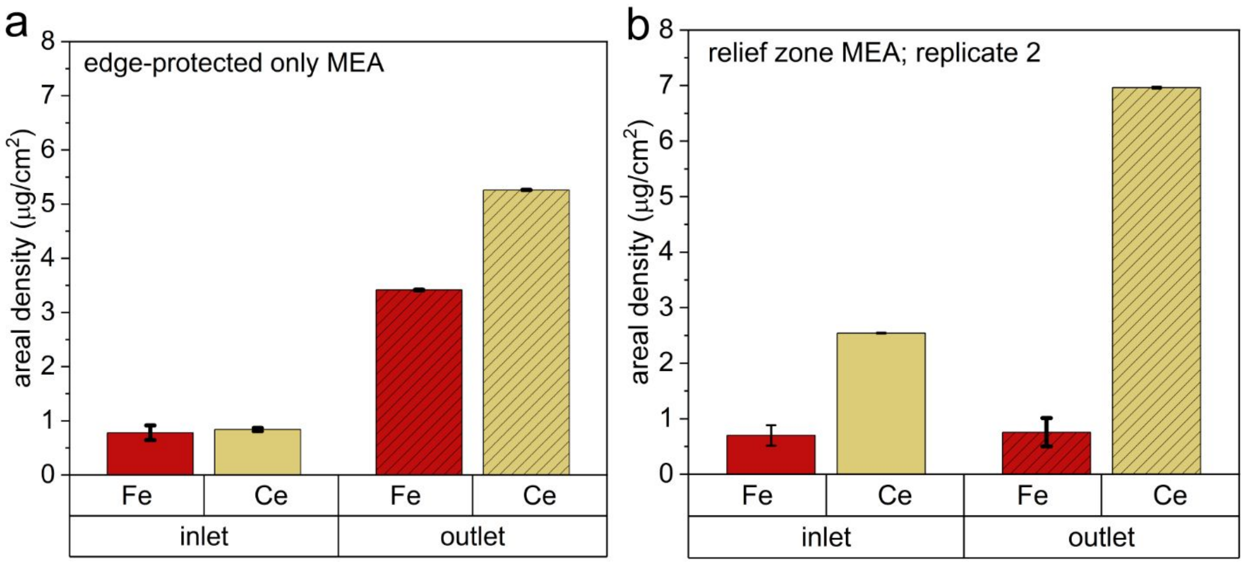

Figure S6. ICP-MS results from the MEAs at EOT. For each MEA, 2 x 2 cm^2 was cut out and soaked in nitric acid to detect iron (Fe) and cerium (Ce) content in the MEA (membrane, anode catalyst layer, and cathode catalyst layer).

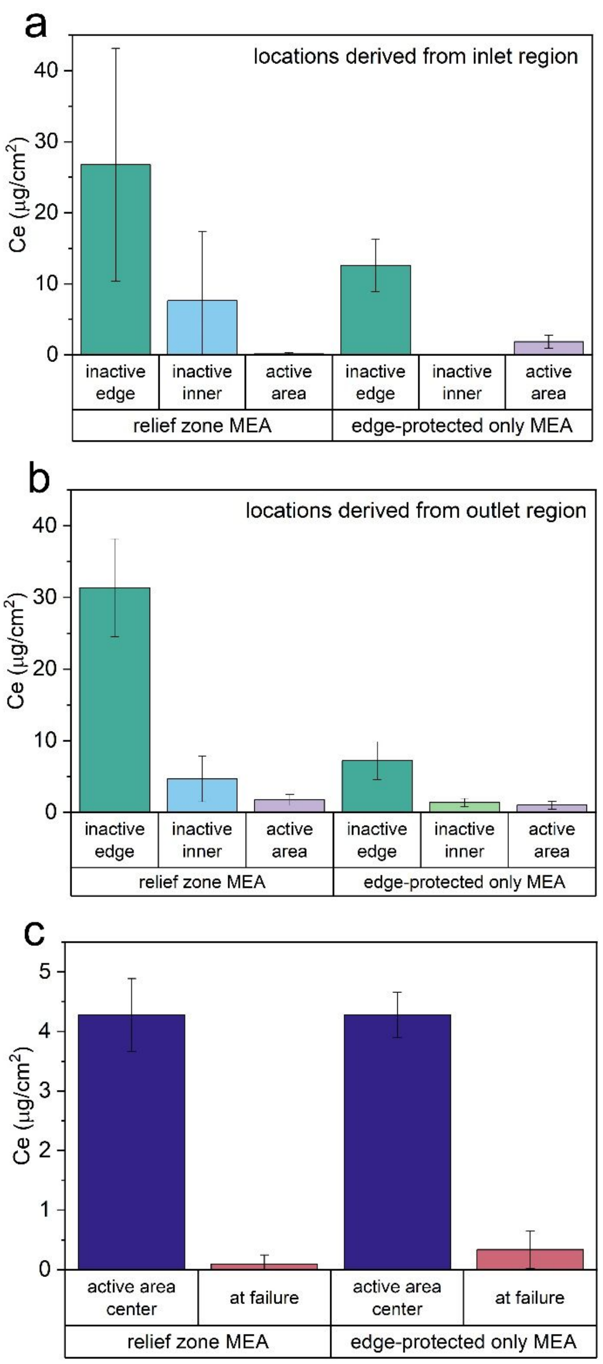

Figure S7. Ce content for the edge-protected only and the relief zone MEAs (replicate 2) measured using XRF. The Ce content was measured in four areas: (a) inlet region, (b) outlet region, (c) center and failure points. For the inlet and outlet regions, the Ce content was measured at the inactive edge (membrane near edge of the hardware), the edge of the active area (just outside the catalyst layers), and at the edge of the active area with catalyst layers.

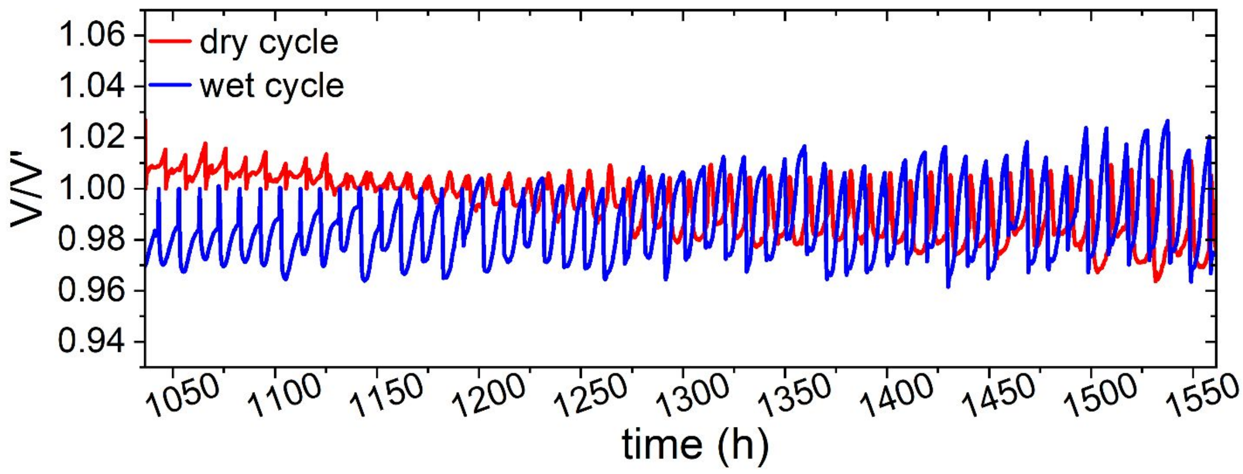

Figure S8. The voltage transient of the relief zone MEA for replicate 1 showing a zoomed-in view of the failure region.

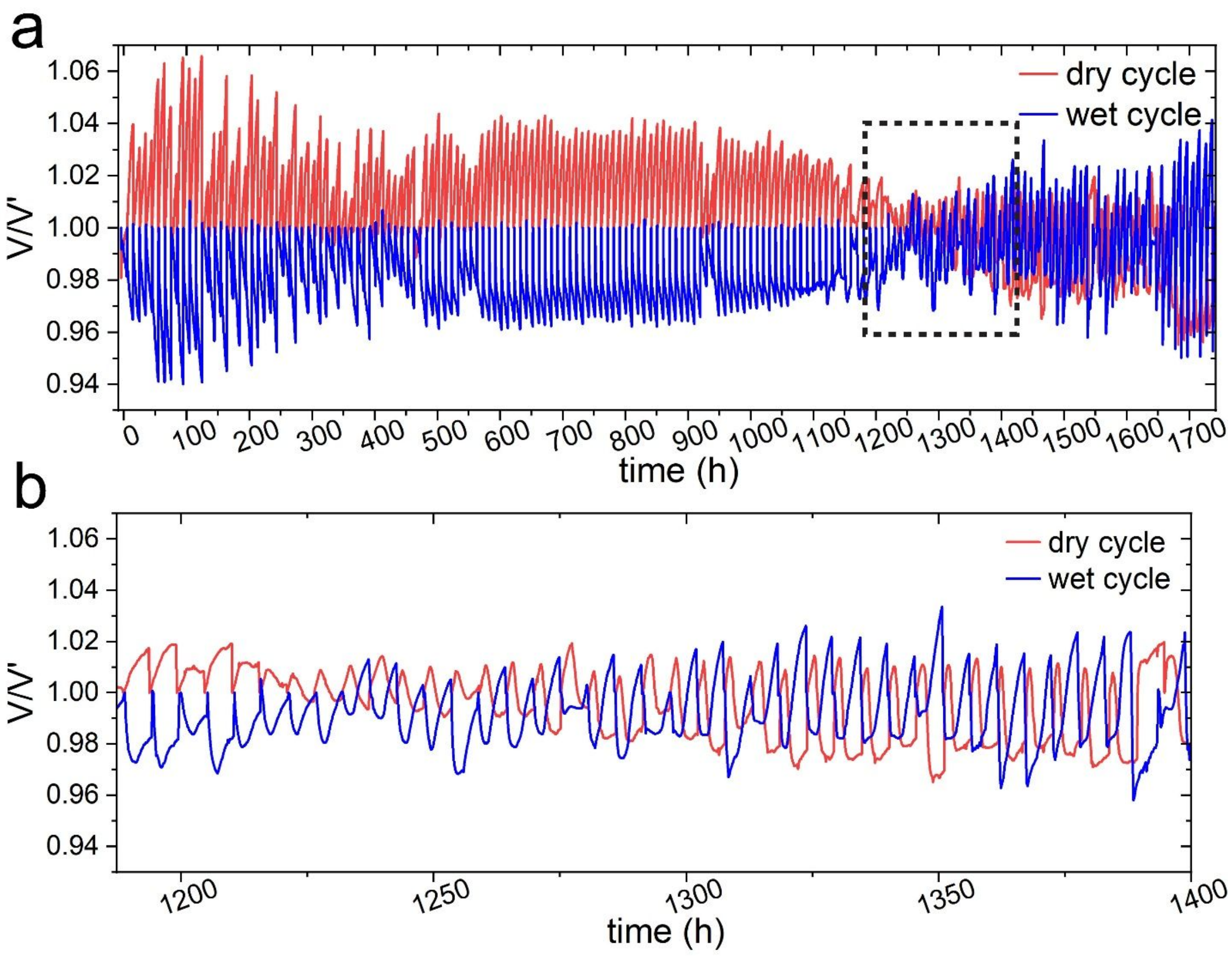

Figure S9. The (a) voltage transient of the relief zone MEA for replicate 2 and (b) a zoomed-in view of the failure region.

References

(1) Taylor, A. K.; Smith, C.; Neyerlin, K. C., Mitigation and Diagnosis of Pin-Hole Formation in Polymer Electrolyte Membrane Fuel Cells. *J. Power Sources* **2023,** *571*, 232971.

(2) Wang, M.; Medina, S.; Ochoa-Lozano, J.; Mauger, S.; Pylypenko, S.; Ulsh, M.; Bender, G., Visualization, Understanding, and Mitigation of Process-Induced-Membrane Irregularities in Gas Diffusion Electrode-Based Polymer Electrolyte Membrane Fuel Cells. *Int. J. Hydrogen Energy* **2021,** *46* (27), 14699-14712.

(3) Wang, M.; Taylor, A. K.; Ochoa-Lozano, J.; Medina, S.; Pfeilsticker, J. R.; Mauger, S. A.; Pylypenko, S.; Ulsh, M.; Bender, G., The Impact of Hot-Press Conditions on the Durability of Polymer Electrolyte Membrane Fuel Cells. *Int. J. Hydrogen Energy* **2025,** *98*, 639-647.

(4) Bender, G.; Felt, W.; Ulsh, M., Detecting and Localizing Failure Points in Proton Exchange Membrane Fuel Cells Using Ir Thermography. *J. Power Sources* **2014,** *253*, 224-229.

(5) James, R. L.; Valentine, S. D.; Liestra, J.; Manufacture or Membrane Electrode Assembly with Edge Protection for PEM Fuel Cells. US Patent 8,470,497: 2006

(6) Wang, M.; Rome, G.; Phillips, A.; Ulsh, M.; Bender, G., Effective Electrode Edge Protection for Proton Exchange Membrane Fuel Cell Drive Cycle Operation. *ECS Trans* **2019,** *92* (8), 351-359.

(7) Kabir, S.; Myers, D. J.; Kariuki, N.; Park, J.; Wang, G.; Baker, A.; Macauley, N.; Mukundan, R.; More, K. L.; Neyerlin, K. C., Elucidating the Dynamic Nature of Fuel Cell Electrodes as a Function of Conditioning: An Ex Situ Material Characterization and in Situ Electrochemical Diagnostic Study. *ACS Appl Mater Interfaces* **2019,** *11* (48), 45016-45030.

(8) Taylor, A. K.; Baez-Cotto, C.; Hu, L.; Smith, C.; Rodriguez-Nazario, A.; Young, J. L.; Mauger, S.; Neyerlin, K. C., The Influence of Electrode Crack Dimensions on the Durability of Polymer Electrolyte Membrane Fuel Cells. *J. Power Sources* **2024**.

(9) Fuel Cell Technical Team; Fuel Cell Roadmap. Sustainability, U.S. Drive Partnership 2013; p 23.

(10) Lai, Y. H.; Rahmoeller, K. M.; Hurst, J. H.; Kukreja, R. S.; Atwan, M.; Maslyn, A. J.; Gittleman, C. S., Accelerated Stress Testing of Fuel Cell Membranes Subjected to Combined Mechanical/Chemical Stressors and Cerium Migration. *J. Electrochem. Soc.* **2018,** *165* (6), F3217-F3229.
